# Acceptability of App-Based Contact Tracing for COVID-19: Cross-Country Survey Study

**DOI:** 10.2196/19857

**Published:** 2020-08-28

**Authors:** Samuel Altmann, Luke Milsom, Hannah Zillessen, Raffaele Blasone, Frederic Gerdon, Ruben Bach, Frauke Kreuter, Daniele Nosenzo, Séverine Toussaert, Johannes Abeler

**Affiliations:** 1 University of Oxford Oxford United Kingdom; 2 University of Mannheim Mannheim Germany; 3 University of Maryland College Park, MD United States; 4 Institute for Employment Research Nürnberg Germany; 5 Aarhus University Aarhus Denmark; 6 University of Nottingham Nottingham United Kingdom

**Keywords:** COVID-19, contact tracing, proximity tracing, app, digital, user acceptability, mHealth, epidemiology

## Abstract

**Background:**

The COVID-19 pandemic is the greatest public health crisis of the last 100 years. Countries have responded with various levels of lockdown to save lives and stop health systems from being overwhelmed. At the same time, lockdowns entail large socioeconomic costs. One exit strategy under consideration is a mobile phone app that traces the close contacts of those infected with COVID-19. Recent research has demonstrated the theoretical effectiveness of this solution in different disease settings. However, concerns have been raised about such apps because of the potential privacy implications. This could limit the acceptability of app-based contact tracing in the general population. As the effectiveness of this approach increases strongly with app uptake, it is crucial to understand public support for this intervention.

**Objective:**

The objective of this study is to investigate the user acceptability of a contact-tracing app in five countries hit by the pandemic.

**Methods:**

We conducted a largescale, multicountry study (N=5995) to measure public support for the digital contact tracing of COVID-19 infections. We ran anonymous online surveys in France, Germany, Italy, the United Kingdom, and the United States. We measured intentions to use a contact-tracing app across different installation regimes (voluntary installation vs automatic installation by mobile phone providers) and studied how these intentions vary across individuals and countries.

**Results:**

We found strong support for the app under both regimes, in all countries, across all subgroups of the population, and irrespective of regional-level COVID-19 mortality rates. We investigated the main factors that may hinder or facilitate uptake and found that concerns about cybersecurity and privacy, together with a lack of trust in the government, are the main barriers to adoption.

**Conclusions:**

Epidemiological evidence shows that app-based contact tracing can suppress the spread of COVID-19 if a high enough proportion of the population uses the app and that it can still reduce the number of infections if uptake is moderate. Our findings show that the willingness to install the app is very high. The available evidence suggests that app-based contact tracing may be a viable approach to control the diffusion of COVID-19.

## Introduction

The COVID-19 pandemic is the greatest public health threat of the last 100 years. In the absence of an effective treatment or vaccination (as of June 2020), the public health response has so far relied on nonpharmaceutical measures to limit the spread of the epidemic, such as physical distancing, case isolation, and manual contact tracing [1]. These measures have not been sufficient to stop the epidemic. Many countries have therefore resorted to partial or full “lockdown” measures to control the epidemic, severely limiting social and economic interactions among their citizens. Although lockdowns may help countries to keep the number of infections under control [2], they come at a great social and economic cost [3-6].

COVID-19 is difficult to trace by traditional methods as COVID-19 cases are infectious 1-2 days before experiencing symptoms and contacts on average become infectious 3-4 days after exposure. The window to achieve containment by manual contact tracing is thus extremely short. Ferretti and colleagues [7] have proposed digital (app-based) contact tracing as an alternative measure to contain the epidemic without the large economic costs of lockdowns. The idea is to use low-energy Bluetooth connections between phones to record the interactions users have with others, particularly those interactions that may pose a higher risk of infection (eg, spending more than 15 minutes within 2 meters of another person). If a user is diagnosed with COVID-19, they can use the app to declare the diagnosis, which then notifies all other users who have come in close contact with the infected person, asking them to isolate at home for 14 days or until they have been tested by the public health authority. The main advantage over traditional (manual) forms of contact tracing is that the app allows instantaneous notification of contacts, which is a key determinant of the effectiveness of case isolation and contact tracing strategies for COVID-19 [7]. Other advantages are that the automatic recording of contacts scales up easily, and avoids the loss of information due to patients’ recall bias and/or imperfect knowledge of the people they have been in contact with.

More and more countries are currently developing various types of contact-tracing apps and several countries have already launched one (eg, Singapore [8], Germany [9], and France [10]). The success of app-based contact tracing, however, critically depends on people’s willingness to use the app. Hinch and colleagues’ [11] epidemic simulation in the United Kingdom shows that the app reduces infections at all levels of uptake but that it is only sufficient to stop the epidemic if approximately 60% of the population use it. It is therefore important to gauge the strength of public support for this approach and to understand the factors that may hinder or facilitate uptake. For instance, since the app would need to trace individuals’ interactions with others, privacy concerns may undermine support and adoption [12]. It is also possible that such a technological solution may not work as well for the less digitally literate share of the population, further increasing the unequal impact of the COVID-19 pandemic within and across countries [13]. In this sense, an “opt-out“ installation policy, where mobile phone providers or Apple and Google [14] would automatically install the app on phones, could maximize uptake. It is unclear, however, whether the public would be willing to support this more intrusive solution.

In light of the many open questions surrounding the viability of app-based contact tracing, we designed a survey to measure public support for this approach in five countries that are currently hit by the COVID-19 pandemic: France, Germany, Italy, the United Kingdom, and the United States. The specific objectives of our study are to (a) assess the overall acceptability among the public of app-based contact tracing under different installation policies (eg, voluntary installation or automatic installation by the government); (b) uncover country-level and individual-level variation in support for the app; and (c) understand the main mechanisms that may facilitate or impede app usage across various subgroups of countries and individuals.

## Methods

### Survey Design

We conducted large online surveys in five countries (France, Germany, Italy, the United Kingdom, and the United States) to measure the acceptability of app-based contact tracing for COVID-19 before apps were introduced in any of the five countries. A complete description of the survey can be found in the Multimedia Appendix 1; here, we provide an overview. At the beginning, after collecting respondents’ informed consent, we described the app, explaining how a general version would function as well as the purpose it would serve (Textbox 1). We abstracted from any details about centralized versus decentralized data storage procedures. Respondents had to pass a comprehension check to proceed further. We then asked respondents how likely they would be to install the app on their phone, if it became available to download voluntarily (“opt-in” installation policy). Respondents were then asked about their main reasons for and against installing the app as well as their compliance with self-isolation requests. Next, we assessed to what degree respondents would be open to an “opt-out” policy, where mobile phone providers would automatically install the app on all phones, but users would be able to uninstall the app at any time. We then collected demographic information and concluded the survey with questions about respondents’ attitudes toward the government under different installation regimes.

**App description in the UK survey.**

“Imagine there was an app that you could install on your mobile phone. This app would automatically alert you if you had been in close contact for at least 15 minutes with someone who was infected with the coronavirus. Such an app does not exist yet in the UK. But we, researchers from the University of Oxford, are interested in understanding what you would think about such an app. [...]

The app would be developed by the NHS. You would need to install the app by simply clicking a link.

Once installed, the app would register which other users are close to you. The app would do this by using Bluetooth and your location.

The app would NOT access your contacts, photos, or other data held on your phone. Only the NHS would have access to the data collected. [...]

If the NHS diagnoses the coronavirus in somebody you have been in close contact with, the app would notify you automatically. The app would give you targeted advice on what to do. It will ask you to self-isolate at home for 14 days or until you have been tested for the virus.

This would be useful since people can infect others even before they have a fever or a cough. Self-isolating would thus protect your family, friends and colleagues from being infected by you. At the same time, only people who were in contact with an infected person would need to self-isolate.

If you had not been in close contact with a confirmed case, then the app would show you an “all clear” message. [...]

If you are diagnosed with coronavirus, the app would notify all people you have been in close contact with, without identifying you to them, and advise them to self-isolate. This would increase the chance of finding all the people you might have infected and help make sure they can keep their loved ones safe as well. If enough people use the app, it will slow down the epidemic and might even stop it entirely.”

*The versions for the other countries differed with regard to the technology used (GPS and Bluetooth vs only Bluetooth) and lockdown restrictions in place generally and tied to app usage specifically. For details, see the Multimedia Appendix 1.*

We kept the survey design as similar as possible across all five countries, with a few exceptions to accommodate country differences with regard to lockdown measures in place at the time of taking the survey. The US survey (deployed last) contained a few additional questions, including robustness checks. See Section A in Multimedia Appendix 1 for more details.

Ethics approval was obtained from the University of Oxford (reference number ECONCIA20-21-06).

No personal information was collected as part of the study.

### Target Population, Sample Size, and Attrition

The surveys were administered between March 20 and April 10, 2020. We recruited respondents through Lucid, an online panel provider. We targeted a sample size of 1000 respondents in each of the four European countries, and 2000 in the United States, with quotas set for the samples to be representative of the overall population in terms of gender, age, and region of residence. A total of 10,375 individuals started the survey and 10,308 consented to participate (participation rate=99%). Out of the people who consented to participate, 6166 passed the comprehension check and started the main questionnaire. After removing incomplete responses and duplicates, we had a sample of 6061 complete and unique responses (completion rate=59%). Finally, we removed 66 respondents who either did not own a mobile phone or did not disclose their gender, leaving us with a final sample of 5995 respondents. To control for the potential effect of our recruitment method, we repeated the German survey with a probability-based sample in an offline recruited online panel. See Section B in Multimedia Appendix 1 for further details on recruitment, filtering and attrition, and the final sample.

### Statistical Analysis

Our main outcome variables measure respondents’ intention to have the app installed on their phone under the two installation regimes (opt-in vs opt-out). The outcomes were measured on a 5-point ordinal scale (opt-in: from *definitely install* to *definitely won’t install*; opt-out: from *definitely keep* to *definitely uninstall*). In our regression analysis, we dichotomized these outcome measures (1=definitely or probably install/keep the app; 0=otherwise).

We used multivariate regression analysis (linear probability models; probit and ordered logit in additional analyses presented in Multimedia Appendix 1) to examine the relationship between intention to install and a number of covariates: age, gender, country, presence of comorbidities (diabetes, high blood pressure, heart or breathing problems), usage of mobile phone outside the house, frequency of social interactions, ability to work from home during the lockdown, ability to obtain sick pay while working from home, trust in national government, and incidence of COVID-19 deaths in a respondent’s region of residence (see Section C.3 in Multimedia Appendix 1 for more details). Table B.2 in Multimedia Appendix 1 presents a summary of these covariates.

## Results

**Figure 1:**
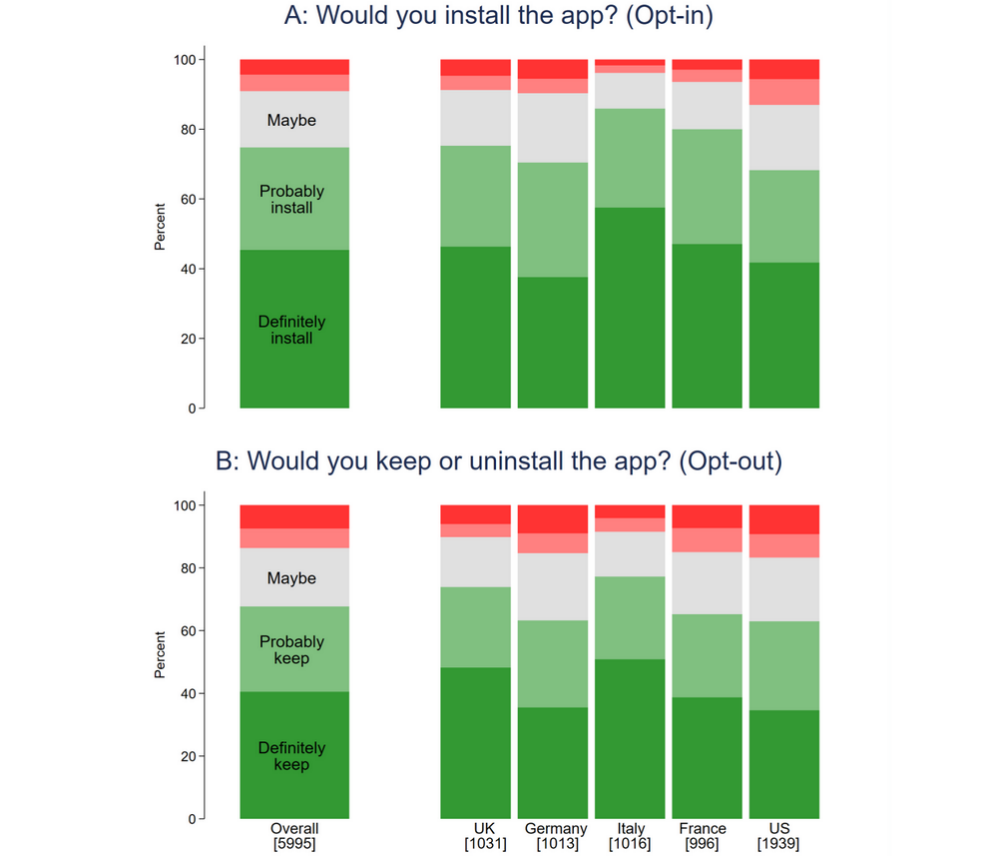
Likelihood of having the app installed, under opt-in and opt-out regimes and by country. Light/dark red bars correspond to *probably/definitely won’t install* in Panel A and *probably/definitely uninstall* in Panel B.

We find broad support for app-based contact tracing. Support is high in all countries, across all subgroups of the population, and under both installation regimes (opt-in and opt-out). Panel A of Figure 1 shows that, under the voluntary (opt-in) installation regime, 4484 out of 5995 respondents (74.8%) across all countries say they would probably or definitely download the contact-tracing app, if it was available. Panel B shows that 4059 out of 5995 respondents (67.7%) say they would probably or definitely keep the app installed on their phone under the automatic (opt-out) installation regime. In both regimes, the share of respondents who say they would not have the app installed on their phone is very small (red portion of the bars in Figure 1).

Support is high in all five countries where we implemented the survey: in each country, at least 68% of respondents say that they would install or keep the app. Moreover, Figures 9–11 in Multimedia Appendix 1 show that support for the app is generally high across various subgroups of the population (eg, across men and women, across different age groups, etc), suggesting widespread acceptability of the app-based contact tracing solution to the COVID-19 pandemic.

Despite the broad and widespread acceptability of the app, we find that support varies systematically across countries and individuals. For instance, Figure 1 shows that Germany and the United States are relatively less supportive of the app compared to the other countries. This is the case both under the opt-in and opt-out regimes. Among individual characteristics, we find that those who have less trust in their national government are more hesitant to have the app installed on their phones (Figure 11 in Multimedia Appendix 1).

**Figure 2:**
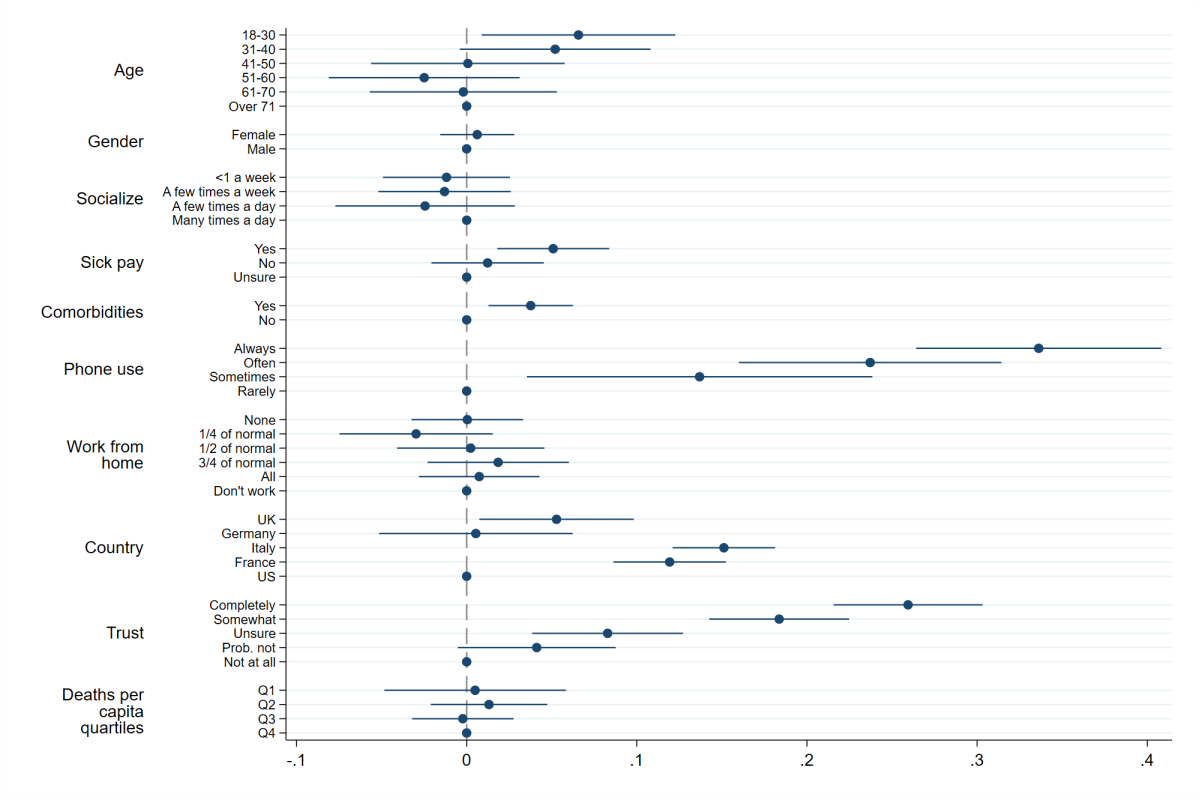
Determinants of stating *definitely install* or *probably install*. Note: the dependent variable is an indicator variable taking the value 1 if a respondent chose *definitely install* or *probably install* when asked whether they would install the app or not, and 0 otherwise. We use a Linear Probability Model. Lines represent 95% CIs calculated with heteroskedasticity-robust standard errors. All coefficients are the result of a single regression and thus display marginal effects. A coefficient of 0.1 implies a respondent who chose this option is 10 percentage points more likely to state they would definitely or probably install the app relative to the base category.

We further explore this heterogeneity using multivariate regression analysis, where we examine the relationship between support for the app and a variety of individual- and country-level covariates. Figure 2 shows the impact that these covariates have on the probability of definitely or probably installing the app under the opt-in regime, using a linear probability model (see Section C.1 in Multimedia Appendix 1 for a similar analysis of the opt-out regime).

The analysis confirms that Germany and the United States are significantly less supportive of the app, especially compared to France and Italy. Taking the two most extreme cases, respondents in Italy are 15.1 percentage points (95% CI 12.1-18.1) more likely to support the app than respondents in the United States. Surprisingly, Figure 2 shows very little correlation between regional-level COVID-19 mortality rates and support for the app.

Among individual-level characteristics, we find that people who carry their phone with them more often are more likely to install the app. Those who always carry their phone with them are 33.6 percentage points (95% CI 26.4-40.8) more likely to support the app than those who carry their phone only rarely. App support is also 3.7 percentage points (95% CI 1.3-6.2) larger among respondents with one or more comorbidities. Moreover, the probability of installing the app increases with trust in the government. People who completely trust the government are 25.9 percentage points (95% CI 21.6-30.3) more likely to install the app than those who do not have any trust in the government.

We found similar results using an ordered logit model, a linear probability model dichotomizing on just *definitely install*, and when using a probit model (Multimedia Appendix 1). Finally, results are also qualitatively similar when considering installation intentions under opt-out rather than opt-in (Figure 8 in Multimedia Appendix 1). Interestingly, under the opt-out regime, trust in government displays an even stronger correlation with the intention to keep the app installed on one’s phone.

We can use the data on respondents’ reasons for or against installing the app to better understand the nature of the observed variation in app support across countries and individuals. A first set of reasons against the app revolved around concerns about government surveillance at the end of the epidemic (mentioned by 2518 out of 5995 respondents, 42%) and cybersecurity (fears that the app could make the phone vulnerable to hackers; 2098/5995, 35%). Respondents also reported that usage of the app may increase feelings of anxiety (1559/5995, 26%), possibly reflecting aversion to feedback about a possible infection. The most frequent reasons in favor of the app were willingness to protect family and friends (4077/5995, 68%), a sense of responsibility toward the community (3177/5995, 53%), and a hope that the app may stop the epidemic (3297/5995, 55%). Figures 16 and 17 in Multimedia Appendix 1 show the relationship between the probability of selecting a particular reason and country- and individual-level characteristics.

Several patterns are of interest. First, we find that, compared to other countries, respondents in Germany and the United States are more likely to mention concerns about government surveillance as one of the reasons against installing the app. In these countries, we also see a larger share of respondents expressing concerns about security of the app, especially compared to Italy and the United Kingdom. Thus, concerns about privacy and security seem to be an important impediment to the adoption of the app, particularly in Germany and the United States.

Among individual-level characteristics, we find that respondents who have less trust in their national government are also more likely to express concerns about government surveillance. This suggests that privacy concerns play a role in the negative relationship between trust in government and the probability of installing the app found in Figure 2. In contrast, we find that frequent usage of mobile phones is related to a stronger perception of the potential benefits of the app: respondents who more often carry their phone with them are more likely to believe that the app would benefit them, by helping them stay healthy and keeping them informed about the risks of infection.

## Discussion

### Principal Findings

In our study, we find high support for app-based contact tracing—irrespective of age, gender, region, or even country of residence. Since the effectiveness of app-based contact tracing crucially depends on a sufficient level of uptake, our findings are encouraging for the prospects of this approach. Although support is high in all countries and subgroups of the population, the data reveal that concerns about cybersecurity and privacy, coupled with trust in government, are important determinants of support. Countries with stronger privacy and security concerns (Germany and the United States) are relatively less supportive of app-based contact tracing. Individuals who have less trust in their national government are also less supportive.

### Implications

The lack of trust in government can have far-reaching implications. Our analysis shows that this factor has a negative effect on people’s intention to install a contact-tracing app on their phones. Furthermore, supplementary analysis (Section C.6 in Multimedia Appendix 1) also shows that people with lower trust in government are more in favor of an opt-in installation policy than an opt-out regime where the government asks mobile phone providers to automatically install the app on all phones. An opt-out regime is likely to translate into higher effective installation rates, for instance, by reducing the negative effects of procrastination or unawareness [15]. However, our data suggests that only governments that enjoy a relatively high level of trust from their citizens may be able to resort to more paternalistic approaches. A policy implication of these findings is that governments should consider delegating the organization of app-based contact tracing to a highly-reputable and transparent public health authority at arm’s length from the government. If the mobile phone’s operating system (eg, iOS or Android) does the contact tracing directly, trust in, for example, Apple or Google, would become more important.

Our results also point toward the need to address privacy and cybersecurity concerns with an app design that respects user personal data as much as possible. Research on the privacy implications of app-based contact tracing, and the potential solutions to these concerns, is currently underway [12,16,17]. Interestingly, however, when we asked our respondents how the data collected by the app should be treated, we find that nearly 60% would consent to making the deidentified data available to research.

### Limitations

Our study has some limitations that we tried to address in different ways. First, respondents recruited online may not be representative of the entire population. In particular, digital literacy and willingness to share data could be higher among such respondents. To ensure that our results do not hinge on our specific sample, we replicated an abridged version of the German survey with a different panel provider that randomly recruits its participants offline. Our results remain almost completely unchanged (Section B.3 in Multimedia Appendix 1).

Second, our survey asked hypothetical questions about future behavior. However, high levels of intended installations may not directly translate into actual installations. While studies often find good correlation between what people declare they would do in surveys and actual behavior [18-22], even in relation to app installations [23-26], many things will have changed between the time of the surveys and when countries eventually introduce the app. For example, at the time of the survey, Italy’s epidemic was close to its peak and the urgency of the situation was very salient. As the epidemic recedes, the perceived need to do something about the epidemic will also recede (eg, [22,27]). Moreover, we made participants aware of the app and explained the app and its potential effects. In reality, many potential users will not be aware of the app, might not engage with the concept, or will not be willing or able to spend the time to find and install the app. More generally, a reported willingness to install is only a necessary first stage to adoption, and our findings about heterogeneity in support point toward specific subgroups of the population that may need stronger encouragements to adoption. We show in Section C.4 of Multimedia Appendix 1 that respondents who would install the app mention far more reasons for its adoption than those who would not install it (but a similar number of reasons against). We show in Section C.9 that respondents in our replication study who did not answer the comprehension questions correctly were less willing to install. Stressing the various benefits of the app, to oneself and others, and explaining the function and purpose of the app may be a particularly effective strategy to foster adoption. Further research will be needed to understand how to translate a person’s willingness to install into the person actually installing the app.

Third, in our survey, we measured support for the general concept of app-based contact tracing, leaving out specific details regarding the implementation, which were not available to us at the time respondents took the survey. One downside of only surveying about the general idea is that it might be harder for respondents to visualize how such a system could work, which may increase hypothetical bias. However, we find that the details we gave about implementation (eg, whether the app uses Bluetooth or GPS) seem to have very little impact on support. This suggests that our general measure of support for app-based contact tracing may be portable across different implementation settings.

Fourth, our results analyzing heterogeneity by age rely on coarse age binning. Such banding is subject to flaws if the sample is not distributed well across bands, which is not possible to verify in our study. Thus, results by age should be read with this limitation in mind.

Finally, our survey respondents were recruited from a specific subset of industrialized Western democracies. Attitudes towards app-based contact tracing may vary across countries with different levels of development and political regimes. It is nevertheless encouraging, in terms of external validity, that we observe a strong similarity in responses across the five countries we sampled and that analogous findings have been reported in ongoing surveys conducted in Australia and Taiwan [28]. In developing countries and among disadvantaged populations, the more limited access to smartphones raises both efficacy and equity issues; the development of low-cost Bluetooth devices with similar functionalities could improve access to digital contact tracing.

### Conclusions

In conclusion, our study shows strong public support for app-based contact tracing to tackle COVID-19. This is an important finding since public support is a necessary condition for the viability of the approach. Further research is needed to gauge the extent to which public support for app-based contact tracing translates into actual app adoption and, more generally, to evaluate its potential for epidemic control.

### Authors’ Contributors

SA and LM were responsible for figures, study design, data collection, data analysis, and writing; HZ for study design, data collection, data analysis, and writing; RB for literature search and data collection; FG and RB for data collection and data analysis; FK for study design, data collection, and data analysis; DN, ST, and JA for study design, data collection, data analysis, and writing.

## Appendix

Multimedia Appendix 1 Online appendix containing additional results, additional information about the samples, and the full questionnaire.
